# DNA methylation analysis of normal colon organoids from familial adenomatous polyposis patients reveals novel insight into colon cancer development

**DOI:** 10.1186/s13148-022-01324-5

**Published:** 2022-08-23

**Authors:** Matthew A. Devall, Stephen Eaton, Mourad Wagdy Ali, Christopher H. Dampier, Daniel Weisenberger, Steven M. Powell, Li Li, Graham Casey

**Affiliations:** 1 Center for Public Health Genomics, University of Virginia, Charlottesville, VA USA; 2 Department of Biochemistry and Molecular Medicine, University of Southern California, Los Angeles, CA USA; 3 Digestive Health Center, University of Virginia, Charlottesville, VA USA; 4 Department of Family Medicine, University of Virginia, Charlottesville, VA USA; 5 Department of Public Health Sciences, University of Virginia, Charlottesville, VA USA

**Keywords:** Familial adenomatous polyposis, Colorectal cancer, Colon organoids, DNA methylation

## Abstract

**Background:**

Familial adenomatous polyposis (FAP) is an inherited colorectal cancer (CRC) syndrome resulting from germ line mutations in the *adenomatous polyposis coli* (*APC*) gene. While FAP accounts for less than 1% of all CRC cases, loss of *APC* expression is seen in > 80% of non-hereditary CRCs. To better understand molecular mechanisms underlying APC-driven CRC, we performed an epigenome-wide analysis of colon organoids derived from normal-appearing colons of FAP patients versus healthy subjects to identify differentially methylated regions (DMRs) that may precede the onset of CRC.

**Results:**

We identified 358 DMRs when comparing colon organoids of FAP patients to those of healthy subjects (FDR < 0.05, |mean beta difference| = 5%). Of these, nearly 50% of DMRs were also differentially methylated in at least one of three CRC tumor and normal adjacent tissue (NAT) cohorts (TCGA-COAD, GSE193535 and ColoCare). Moreover, 27 of the DMRs mapped to CRC genome-wide association study (GWAS) loci. We provide evidence suggesting that some of these DMRs led to significant differences in gene expression of adjacent genes using quantitative PCR. For example, we identified significantly greater expression of five genes: *Kazal-type serine peptidase inhibitor domain 1* (*KAZALD1*, *P* = 0.032), *F-Box and leucine-rich repeat protein 8* (*FBXL8, P* = 0.036), *TRIM31 antisense RNA 1* (*TRIM31-AS1*, *P* = 0.036), *Fas apoptotic inhibitory molecule 2* (*FAIM2*, *P* = 0.049) and (*Collagen beta (1–0)galactosyltransferase 2* (*COLGALT2*, *P* = 0.049). Importantly, both *FBXL8* and *TRIM31-AS1* were also significantly differentially expressed in TCGA-COAD tumor versus matched NAT, supporting a role for these genes in CRC tumor development.

**Conclusions:**

We performed the first DNA methylome-wide analysis of normal colon organoids derived from FAP patients compared to those of healthy subjects. Our results reveal that normal colon organoids from FAP patients exhibit extensive epigenetic differences compared to those of healthy subjects that appear similar to those exhibited in CRC tumor. Our analyses therefore identify DMRs and candidate target genes that are potentially important in CRC tumor development in FAP, with potential implications for non-hereditary CRC.

**Supplementary Information:**

The online version contains supplementary material available at 10.1186/s13148-022-01324-5.

## Background

Familial adenomatous polyposis (FAP) is an inherited colorectal cancer (CRC) syndrome. Patients with classic FAP typically develop hundreds of adenomatous polyps in their late teens or early twenties [1]. If left untreated, the development of CRC is almost inevitable, with FAP patients developing cancer, on average, by 39 years of age [2].

FAP is driven by inherited or de novo germ line inactivating mutations of the *adenomatous polyposis coli* (*APC*) gene. While FAP accounts for less than 1% of all colorectal cancers (CRC) [3], loss of *APC* expression is seen in more than 80% of CRC cases, either through somatic mutation or promoter hypermethylation [4]. APC functions as a negative regulator of the Wnt/β-catenin signaling pathway [5] through an APC/AXIN/GSK3 β-catenin destruction complex. Inactivation of *APC* results in loss of this complex, stabilization of β-catenin, translocation of β-catenin into the nucleus and an eventual increase in cellular proliferation [6]. While a number of important contributions to CRC biology have been made through the study of *Apc* knockout mouse and rat models [7], significant phenotype variability has been observed not only when comparing differing models, but also through comparisons of the same model in different laboratories [4, 8]. Furthermore, while *APC* mutations in FAP lead to a severe polyposis largely restricted to the colon, *Apc* mouse models are not fully reflective of human disease and lead to tumors located primarily within the small intestine [4, 9, 10]. As such, a number of obstacles still limit our understanding of the early molecular events in FAP cancer development. However, the normal colon organoid model system offers a novel and promising approach that may overcome some of these limitations and enable the studying of those early events [11].

In recent years, our group has successfully employed the normal colon organoid model [12] to study the role of CRC risk factors in colon epithelial cell biology [13–16]. The colon organoid system serves as a model of the epithelial cells of the colonic crypt, particularly the colon crypt stem cell niche. These cells are hypothesized to serve as the origin for CRC tumorigenesis. This system has become an increasingly popular tool to understand basic biology of gastrointestinal cancers, as well as predicting individual response to treatment therapies [11].

DNA methylation is a reversible epigenetic modification, and aberrant DNA methylation therefore has the potential to serve not only as a biomarker for disease, but also as a potential druggable target. In CRC, DNA methylation studies have led to the development of widely used biomarkers [17] and aberrant patterns of DNA methylation have also been suggested to play an important role in CRC development and pathogenesis [18]. Importantly, previous research has shown that the colon organoid model retains regional patterns of DNA methylation and gene expression following their establishment [19–21] and that organoids may even reflect patterns of epigenetic aging observed in both the colon mucosa and crypts from which they were derived [22]. Therefore, investigating the role of DNA methylation in an organoid model system of FAP subjects who are highly likely to develop CRC has the potential to provide unique molecular insight into the initiation of CRC in these individuals.

To develop a better understanding of the biology underlying risk for CRC posed by germ line *APC* mutations we performed DNA methylation analysis (Illumina Infinium MethylationEPIC, herein EPIC array) of colon organoids derived from normal-appearing colons of FAP subjects (*n* = 7) and healthy individuals (*n* = 16). We identified a large number (*n* = 358) of differentially methylated regions (DMRs) between colon organoids of FAP and healthy subjects. In an attempt to relate these differences to non-hereditary forms of CRC, we compared our findings between FAP and healthy individuals to those between tumor and normal adjacent tissues (NAT) in three independent, publicly available, non-hereditary CRC cohorts [18, 23, 24]. Finally, we provided evidence to support a functional role of these DMRs by using quantitative PCR (qPCR) to investigate expression differences of a subset of the putative target genes. Our studies provide potential insight into early tumorigenic events in FAP that may also be relevant to the development of non-hereditary forms of CRC.

## Results

### Analysis of normal colon organoids from FAP and healthy subjects reveals cancer-related DNA methylation differences

Colon organoids derived from normal-appearing colons of FAP patients and healthy subjects were grown in complex media, as described previously [13–16]. All colon organoids used within this study were derived from distal (left) colon biopsies. We performed epigenetic clock analysis using epiTOC2 [25] and the Horvath age clock [26]. A significant increase in age acceleration residual [26] was observed in FAP colon organoids (*P* = 0.031). While no differences in mitotic age, HypoClock or pcgtAge were observed, a significant increase in the average lifetime intrinsic rate of stem cell division was identified in FAP organoids (*P* = 0.018) when taking age at colonoscopy into account (Additional file 1: Fig. S1). A higher proliferative rate and mean level of mitosis have previously been reported in colon crypts of FAP patients [27]. DMR analysis [28] was performed between the two groups while accounting for age at the time of colonoscopy and the individual’s reported biological sex (Additional file 2: Table S1). This led to the identification of 358 DMRs across 439 unique genes, of which approximately half (52.79%) were hypermethylated in FAP. The most significant of these findings corresponded to the genomic region containing *Deleted in lymphatic leukemia 1* (*DLEU1*, FDR = 3.08E^−22^), which was hypomethylated in FAP and has previously been described as a tumor suppressor gene in non-CRC cancers [29]. Notably, there was an absence of differential methylation at the *APC* gene locus. Pathway analysis of the DMRs revealed nominal enrichments for 307 gene ontology (GO) terms (*P* < 0.01), of which 16 survived FDR correction at 5%. These included differences such as “homophilic cell adhesion via plasma membrane adhesion molecules” (FDR = 0.011), “cell development” (FDR = 0.035) and “cell–cell adhesion via plasma-membrane adhesion molecules” (FDR = 0.035), while nominal enrichments for “negative regulation of cell differentiation" (*P* = 2.36E^−04^), "regulation of cell population proliferation” (*P* = 3.41E^−03^) and “digestive tract morphogenesis” (*P* = 4.09E^−03^) were also observed. Despite this, we observed no significant differences in cell viability or proliferation between organoids of FAP and healthy subjects. We observed no gross morphological differences in colon organoids of FAP patients compared to those of healthy subjects (Additional file 3: Fig. S2).

### Relationship between findings in FAP organoids and CRC biology

**Fig. 1:**
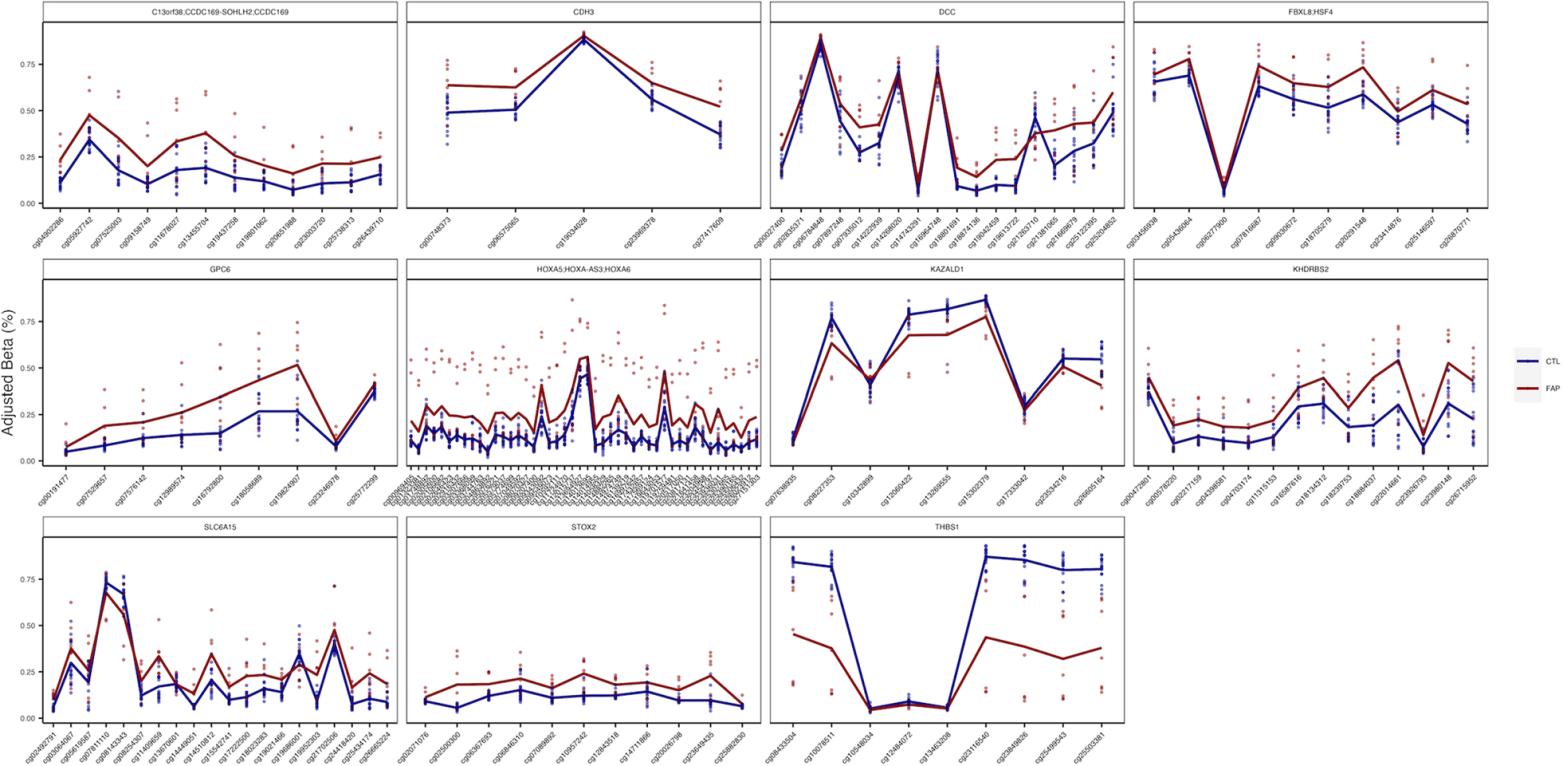
Overview of significant DMRs that were present in FAP and all three cancer cohorts. Adjusted beta values (%) were plotted for each CpG and sample across the region of interest

We employed two approaches to better understand the relevance of the observed DMRs to CRC biology. First, more than 140 inherited genetic variants have been associated with CRC risk through genome-wide association studies (GWAS) [30]. By intersecting DMRs within 1 Mb across each CRC GWAS locus, we found that 27 DMRs mapped to 29 GWAS loci (Table 1), implicating these genes in CRC tumorigenesis. To approximate the relative significance of this overlap, we performed an analysis of individual CpG sites associated with FAP. Fisher’s test for enrichment revealed that this overlap was not significant. Second, we downloaded and processed publicly available DNA methylation data from matched CRC tumor and NAT from three independent CRC tumor cohorts: TCGA-COAD (*n* = 36 pairs), GSE193535 (*n* = 47 pairs) and ColoCare (*n* = 78 pairs). DNA methylation analysis was performed on each of these cohorts independently using DMRcate [28]. We found that 40.08% (146) of FAP DMRs were significant in at least two cancer cohorts and displayed the same direction of effect in CRC tumor versus NAT. This included a subset of nine DMRs that mapped to CRC GWAS loci (Table 1, bold font). An additional 8.94% [32] were identified in at least one of the three cancer cohorts. Further, 112 DMRs of the 358 FAP DMRs were present across all three cancer cohorts (31.29%, Fig. 1), with the same direction of effect. Fisher’s test for enrichment revealed that a greater number of significant FAP related CpG sites were identified than expected by chance in our individual analyses of TCGA-COAD (*P* = 1.19E^−20^), GSE193535 (*P* = 1.22E^−16^) and ColoCare (*P* = 7.64E^−21^). While most of the DMRs were concordant, 10.36% [37] of the 358 DMRs displayed discordance for direction of effect in at least two cancer cohorts and FAP patients. This included *DLEU1*, which was hypomethylated in FAP versus normal colon organoids, but significantly hypermethylated in all three tumor vs NAT analyses (Additional file 4: Table S2).

**Table 1:** Summary of DMR’s identified in the analysis of FAP versus healthy colon organoids that overlapped with CRC GWAS SNPs

Genomic position (Number of CpGs in region)	*P*	FDR	Mean difference (Beta)	Overlapping genes	SNP
chr13: 78492568–78494462 [41]	4.90E−21	9.43E−19	− 0.117	*RNF219-AS1, EDNRB*	rs1330889
chr5: 135415190–135416613 [16]	3.48E−20	4.46E−18	− 0.246	*VTRNA2-1*	rs4976270
**chr13: 36871646–36872346 **[12]	**4.47E−15**	**2.15E−13**	**0.135**	***SOHLH2, CCDC169-SOHLH2, CCDC169***	**rs7333607**
chr10: 50602990–50604518 [14]	4.75E−10	8.70E−09	− 0.085	*DRGX*	rs10821907
**chr16: 68676451–68677364 **[8]	**1.24E−09**	**2.08E−08**	**0.123**	***CDH3***	**rs9924886**
chr6: 31894831–31895598 [9]	2.04E−09	3.27E−08	− 0.105	*C2, CFB*	rs2516420, rs3830041
**chr10: 102821427–102822249 **[9]	**2.05E−08**	**2.25E−07**	** − 0.086**	***KAZALD1***	rs4919687
chr12: 50614713–50616779 [9]	5.49E−07	3.77E−06	− 0.093	*RP3-405J10.4, LIMA1*	rs12372718
chr12: 115124584–115126061 [8]	7.46E−06	3.26E−05	− 0.136	*NA*	rs7300312
chr5: 126408756–126410348 [14]	8.17E−05	2.35E−04	− 0.142	*C5orf63*	rs12659017
chr6: 32115979–32116963 [19]	9.69E−05	2.70E−04	− 0.083	*PRRT1*	rs3830041, rs2516420
chr1: 109849705–109850837 [8]	1.50E−04	3.94E−04	− 0.072	*NA*	rs2938616
chr6: 35108605–35109398 [12]	3.73E−04	8.08E−04	0.066	*TCP11*	rs16878812
chr10: 99734513–99735202 [7]	4.99E−04	1.04E−03	0.080	*CRTAC1*	rs10786560, rs11190164
**chr6: 29795595–29796614 **[11]	**7.37E−04**	**1.40E−03**	**0.108**	***HLA-G, HCG4P8***	**rs1476570**
chr15: 67390372–67391147 [7]	7.42E−04	1.40E−03	− 0.132	*SMAD3*	rs56324967, rs745213
**chr6: 30079139–30079662 **[13]	**7.61E−04**	**1.42E−03**	** − 0.135**	***TRIM31-AS1, TRIM31***	**rs3131043**
chr3: 113160071–113160821 [13]	2.76E−03	4.25E−03	0.051	*WDR52*	rs13086367, rs12635946, rs72942485
**chr12: 50297581–50298198 **[7]	**2.81E−03**	**4.32E−03**	**0.064**	***FAIM2***	**rs12372718**
chr19: 58446600–58446988 [10]	2.92E−03	4.45E−03	− 0.073	*ZNF418*	rs73068325
chr12: 95945082–95945927 [10]	4.76E−03	6.76E−03	− 0.069	*USP44*	rs11108175
chr6: 31276088–31276797 [13]	6.61E−03	8.93E−03	− 0.065	*XXbac-BPG248L24.10*	rs3131043, rs116353863, rs116685461
**chr20: 62795464–62796178 **[9]	**8.16E−03**	**0.011**	** − 0.073**	***MYT1***	**rs1741640**
**chr11: 101454317–101454996 **[12]	**9.46E−03**	**0.012**	**0.106**	***TRPC6***	**rs2186607**
**chr6: 31733889–31734232 **[9]	**0.016**	**0.020**	**0.073**	***VWA7***	**rs116685461, rs2516420**
chr11: 111385338–111385778 [7]	0.026	0.029	− 0.070	*C11orf88, RP11-794P6.6*	rs3087967
chr5: 134914923–134915088 [7]	0.038	0.042	− 0.074	*CTC-321K16.1, CXCL14*	rs4976270

DMR was not present in any analysis

Positive mean beta differences correspond to DNA hypermethylation of that region in FAP colon organoids. Bold font indicates genes that were also present in at least two analyses of tumor versus NAT, with the same direction of effect

Pathway analysis revealed that 152 of 305 GO pathways identified in our analysis of FAP versus healthy subjects were at least nominally enriched (*P* < 0.01) in all three cancer cohorts (Additional file 5: Table S3), indicating a strong concordance between pathway level events occurring in normal colon organoids of FAP patients and in CRC tumors. Indeed, 15 of the 16 FDR-corrected FAP pathways also survived FDR corrections in both cancer cohorts, including enrichments at all six pathways highlighted in FAP colon organoids.

### qPCR of target genes identifies *FBXL8* as a potentially important target in CRC tumor development

To determine whether the observed differences in DNA methylation potentially drive gene expression, we performed qPCR on a subset of the corresponding putative target genes in colon organoids of five FAP patients and five healthy subjects (Additional file 6: Table S4). Genes were selected if they corresponded to a DMR and were replicated in at least two of three cancer cohorts at the DNA methylation level. For DMRs that extended over multiple genes, a site-specific analysis was performed across that locus to determine the location of the most significant difference (data not shown). The gene that corresponded to this location (or the closest gene) was selected for targeted gene expression analysis. Preference was also given to genes that mapped within CRC GWAS loci (*Cadherin 3* (*CDH3*), *Fas apoptotic inhibitory molecule 2* (*FAIM2*), *von Willebrand factor A domain containing 7* (*VWA7*), *Kazal-type serine peptidase inhibitor domain 1* (*KAZALD1*) and *TRIM31 antisense RNA 1* (*TRIM31-AS1*)). We found that five of the 12 genes assayed displayed significant differences in gene expression in colon organoids of FAP compared to healthy subjects (*P* < 0.05). However, of these five, only *TRIM31-AS1* (*P* = 0.036) and *FBXL8* (*P* = 0.036) also showed higher expression in tumors versus NAT in the TCGA-COAD cohort (*P* = 1.80E^−03^ and *P* = 1.05E^−03^, respectively). RNA-sequencing data from TCGA-COAD revealed significant differences in the expression of *CDH3* (*P* = 1.19E^−176^), *Homeobox A5* ((*HOXA5*), *P* = 3.20E^−03^) and *Coiled-coil domain containing 170* ((*CCDC170*), *P* = 0.024), but only expression of *CCDC170* neared significance in our FAP qPCR analysis (*P* = 0.14). Further, expression of *Collagen beta (1–0)galactosyltransferase 2* (*COLGALT2*) was significantly increased in our FAP colon organoid dataset (*P* = 0.049), though the opposite was true in TCGA-COAD (*P* = 1.05E^−04^). Analysis of gene expression was not performed in ColoCare, which used microarray rather than sequencing technology to assess transcriptional differences. No gene expression data were available for the GSE193535 cohort.

Survival analysis of *FBXL8*, performed in GEPIA2 [31], revealed that high *FBXL8* expression trended with reduced overall survival (*P* = 0.054) and disease-free survival (*P* = 0.073) in TCGA-COAD. Further, a negative correlation (*R* =  − 0.27, *P* = 9.80E^−07^) between expression of this gene and *APC* expression was observed in sigmoid and transverse colon locations [31, 32]. No significant differences in survival were seen for *TRIM31-AS1* in the TCGA-COAD cohort.

## Discussion

Few epigenome-wide studies have interrogated the molecular events occurring in normal colons of FAP patients despite the near certainty that FAP patients develop CRC. Indeed, a search of relevant, public databases for DNA methylation datasets (Gene Expression Omnibus (GEO), ArrayExpress) revealed only one small study with available epigenome-wide data (Illumina HM450 or EPIC). This study was limited to adenomas and tumor samples of two FAP patients [33]. Inactivation of or mutations in the *APC* gene have been extensively studied in the field of CRC [4]. Our identification of differences in DNA methylation that may contribute to, or drive, the FAP phenotype has the potential to offer significant insight into both FAP and non-hereditary forms of CRC. Further research should consider the use of the colon organoid model for such analysis. The human colon organoid system employed here does not suffer from species-specific differences [4] and is unlikely to be burdened by the effects of extensive somatic mutations present in tumor samples. Instead, by serving as a model of the colon crypt, the colon organoid system employed here is naïve to both stromal and immune cell compartments. It has been hypothesized that the cells of the stem cell niche act as the origin for CRC [34]. Thus, analysis of important driver mutations for CRC development in this epithelial cell compartment has the potential to better identify DMRs relevant to early CRC biology. Thus, the identification of downstream molecular events related to *APC* has the potential to lead to the identification of genes critical to tumor development [35], some of which may be modifiable through drug targeting [36] or gene editing [37].

A primary strength of this study lies in our effort to replicate findings at the DMR level using three cancer cohorts of differing ethnicities. Indeed, of the 358 DMRs that we identified in colon organoids of FAP versus healthy individuals, almost 50% were identified in at least one CRC tumor/NAT cohort, while 112 were consistent across all three. By extending our approach to the investigation of gene expression, we were able to identify the potential, functional implications of these consistent DMRs. For example, *FBXL8* showed significantly higher expression in both colon organoids of FAP versus healthy subjects and in TCGA-COAD tumor versus NAT. We also note that expression of *FBXL8* and *APC* is negatively correlated in normal tissue [31, 32], which may be important given the role of *APC* in FAP development. Few studies on *FBXL8* have been undertaken in CRC [38]; however, this gene has been reported to be significantly upregulated in breast cancer [39], mirroring our analysis of the TCGA-COAD dataset. The same study also showed that *FBXL8* may be a novel anti-apoptosis factor and was positively correlated with higher breast cancer stage. This result is also somewhat in line with our findings, where increased *FBXL8* expression trended with a reduction in survival. Additional mechanistic studies are warranted to determine whether *FBXL8* may represent a novel target for prevention/treatment of CRC.

Genes prioritized for gene expression analysis were considered based on DMRs being identified in organoids of FAP versus healthy subjects and in tumor versus NAT in TCGA-COAD and at least one additional CRC cohorts. Given that differential DNA methylation occurred at overlapping loci across cohorts, it was expected that similar gene expression differences would also be identified by qPCR in FAP versus healthy colon organoids and RNA-sequencing of TCGA-COAD. However, not all significant gene expression differences were consistent when comparing across these two analyses. For example, while a significant DMR was found at *CCDC170* in FAP and in both TCGA-COAD and ColoCare, *CCDC170* did not show significantly higher expression in colon organoids of FAP versus healthy subjects by qPCR, despite *CCDC170* expression being significantly greater in TCGA-COAD tumors than NAT. The regulation of gene expression is highly complex and not entirely dependent upon local alterations to DNA methylation. It is therefore possible that other nearby loci are also acting to impact gene expression of these targets. We expect that analysis of additional FAP subjects, an incorporation of a transcriptome-wide assessment and the employment of methods for integration of multiple omic layers may help clarify the role of this gene and others identified in our study, in FAP and CRC.

To further understand the potential role of the observed DMRs identified in FAP organoids in non-hereditary CRC tumorigenesis we examined the overlap of DMRs with CRC GWAS loci. While over 140 genomic loci have been associated with CRC risk, few have led to the identification of the functional SNP and downstream target gene (s). This has led to a severe roadblock to progress in our understanding of complex genetic diseases such as CRC and understanding of early events in CRC tumorigenesis. Of the 27 DMRs identified that mapped to GWAS loci, nine were also identified in at least two of three CRC tumor versus NAT datasets, with the same direction of effect. A DMR adjacent to *TRIM31-AS1* and *TRIM31* was found to be significantly hypomethylated in FAP versus healthy and in all three CRC tumor datasets. Both of these genes map to CRC GWAS loci. Interestingly, *TRIM31-AS1* displayed higher expression in colon organoids of FAP compared to healthy subjects and in TCGA-COAD tumors versus NAT. Given that a primary function of antisense RNA is to bind protein coding mRNA and block translation, increased expression of *TRIM31-AS1* may be important in protein regulation, although a role for this specific antisense RNA has not been determined in CRC. These data strongly implicate these DMRs (and the genes that correspond to them) in early tumorigenesis not only in FAP subjects but also in non-hereditary CRC.

For each DMR analysis, we extended the scope of our findings beyond the single-gene approach to determine whether differences in DNA methylation were enriched for genes found within specific GO terms. We identified over 300 GO terms that were nominally associated with differences in DNA methylation in colon organoids from FAP versus healthy subjects. Importantly, as with single DMR analysis, many of these differences were also seen when comparing CRC tumor and NAT, suggesting that signaling pathway aberrations are somewhat consistent between FAP normal crypt epithelium and tumor samples. Of note, many of these enriched terms were relevant to cell migration, differentiation and proliferation, all of which are dysregulated and contribute to CRC. Interestingly, we previously reported irregular patterns of leucine-rich repeat containing G protein-coupled receptor 5 (LGR5)-positive stem cells that were not limited to the base of the colonic crypt of FAP patients, indicating a dysregulation of the stem cell niche in normal colon of FAP subjects [40]. Further exploration into the drivers of these aberrant processes has the potential to reveal relevant clinical targets for CRC prevention and treatment.

We recognize a number of limitations to our study. For example, it is feasible that the observed differences in methylated regions of DNA between normal colon organoids of FAP and healthy subjects represent inter-individual differences in lifestyle, particularly given the relatively small size of our pilot study. In an attempt to mitigate this possibility, we prioritized only DMRs/genes that were also present in at least two CRC tumor/NAT datasets for further consideration. However, in doing so, we limit the scope of our study only to DMRs that share molecular events between FAP and non-hereditary CRC. As such, the functional relevance of DMRs to FAP such as *DLEU1* hypomethylation was not considered. We also performed our secondary analysis using a less dense array platform (Illumina HM450 vs EPIC). Furthermore, we removed probes that have been implicated as race-related or those considered to be driven by somatic mutations using SeSAMe [41]. While this allowed for our findings to be contextualized in the framework of CRC, false negatives may have been introduced if multiple probes were removed during these steps, or if the DMR was not present on the Illumina HM450 array. Our use of the colon organoid system reduces the confounding effects of cellular heterogeneity, but this may mask true biological signal of DNA methylation within relevant cell types (e.g., stem cells). However, we note that the use of colon organoids does not address the influence of other cells of the colon such as immune cells, which are also known to be important to CRC establishment [11]. Future studies should consider how colon organoid co-culture methods may be better adapted for the analysis of continual CRC development. Previous studies have demonstrated that long-term culture of colon organoids (12–14 months) leads to alterations in the DNA methylome at sites relevant to CRC [42]. While possible that some short-term culture effects may impact the DNA methylome of organoids grown within our dataset, steps were taken to mitigate this: organoids were grown together; passage number remained low (typically between eight and ten). There were also some statistical limitations to our study. For example, we identified overlapping DMRs between FAP colon organoids and CRC tumors as well as with GWAS-related loci. However, it is unclear whether the extent of this overlap is significant. In part, this is driven by unequal spacing of probes on, and across, the two arrays considered here. Further, the number of co-methylated DMRs present for use as an appropriate background set remains unclear and would likely be of variable length. To overcome this limitation, we performed Fisher’s exact test on site-specific analysis of FAP colon organoids and compared the overlap observed across analyses to that expected by chance. We observed a consistent enrichment for CRC tumor-related, DNA methylation differences and the differentially methylated CpGs identified within our FAP analysis. This adds weight to our belief that the FAP colon organoid model is a useful model for early CRC-related events. The lack of enrichment for CRC GWAS loci is not surprising given the limited number of DMRs found within 1 Mb of the CRC GWAS index SNP, but larger, future studies with more power should aim to revisit this association.

In conclusion, we performed the first DNA methylation array analysis of colon organoids derived from normal appearing colon of FAP and healthy subjects and systematically leverage findings against publicly available data to identify DMRs and putative corresponding genes that may be important not only to FAP biology, but also non-hereditary CRC tumor development. We believe that expansion of the novel framework that we describe can be used to better understand early molecular events in CRC tumor biology, which has the potential to lead to insight into novel druggable targets.

## Methods

### Patient selection

Healthy subjects (*n* = 16) undergoing standard of care colonoscopy were recruited at the University of Virginia (UVA) alongside FAP patients (*n* = 7) who were undergoing surveillance colonoscopies. All healthy subjects included in the study presented with 3 or fewer polyps and no personal or immediate family history of CRC. FAP was defined by clinical presentation and/or genetic mutation. FAP and healthy colon organoids displayed no statistically significant differences for age, biological sex or smoking status, though five of the 16 healthy subjects were current or previous smokers. All subjects were self-reported to be of White ethnicity.

### Biopsy collection and establishment of colon organoids

Colon organoids from healthy and FAP subjects were established from biopsies taken at colonoscopy using standard forceps. For healthy subjects, four biopsies were taken immediately distal to the hepatic flexure (right colon) and four immediately distal to the splenic flexure (left colon) for the establishment of organoids. For FAP patients, biopsies were limited by the normal colon that was available. To reduce the potential of colon location-specific effects on DNA methylation, this study included organoids derived from the left colon only. FAP and healthy colon organoids were grown at the same time, under the same conditions to minimize the effects of batch on our dataset. Our procedure for establishing colon organoids has been described elsewhere [13–16]. All colon organoids considered for this study were between passages eight-ten.

### Cellular imaging and viability analysis

For viability analysis, Matrigel was scratched off and resuspended in fresh culture medium, described elsewhere [13–16]. Organoids were broken down into single cells by washing each well with 500 μL DPBS (Gibco, ref: 10,010–031) and 10 μM Y27632 (R&D Systems ref: 1254) and transfer the additional 500 μL/well to the same tubes. Organoids were centrifuged at 300 × *g*, 5 min, 4 °C and the supernatant was aspirated, being careful not to disturb the Matrigel layer. They were then resuspended in 1 mL Accutase (Corning, ref: 25–058-CL) with 10 μM Y27632 and incubated for 15 min at 37 °C. They were then neutralized with 2 mL wash medium (DMEM/F12 (ref: 12634-010), 10% FBS (Gibco ref# 16000-044), 2 mM L-Glutamine (Cellgro, ref: 25–005-Cl), 1X GlutaMAX (Gibco, ref: 35050-061), 10 mM HEPES (Gibco, ref: 15630-080) and 100 U/mL Pen-Strep (Gibco, ref: 15140-122)) and dispersed eight times with a 1-mL syringe/25G needle. Following this, organoids were centrifuged at 300× *g*, 5 min, 4 °C and the pellet was resuspended in 1 mL TrypLE Express (Gibco, ref: 12604013) with 10 μM Y27632, where it was incubated for 15 min at 37 °C before being neutralized with 2 mL wash medium coupled with 8× dispersion using a 1-mL syringe/25G needle. Cells were then centrifuged at 300× *g*, 5 min, 4 °C and resuspended in 500 μL wash medium to prepare for cell counting. Cells were plated at a density of 20 K cells per well (30 μL/well). They were then incubated for 15 min at 37 °C and fed 500 μL/well culture medium. Cells were fed every 2–3 days. Imaging was taken on day 9 of organoid growth for each sample using a Lumenera Infinity2-2C 2.0 Megapixel CCD Color Camera (cat. #95107) and Infinity Analyze software at 100× magnification. Cell viability and counts were taken before and after the experiment using a Countess II Automated Cell Counter (Invitrogen: AMQAX1000).

### DNA extraction and bisulfite treatment

Genomic DNA was extracted using the Qiagen UCP DNA kit (Catalog No: 56204; Qiagen; Hilden, Germany) with few exceptions. Extraction of FAP and healthy colon organoids was performed at the same time in an attempt to minimize batch effects. For elution, a 5-min final incubation of Buffer AUE was preferred to increase yield. Further, the elution step was carried out twice using two volumes of 25 μL. DNA quality was assessed using gel electrophoresis to ensure that DNA was not heavily fragmented. Genomic DNA samples were bisulfite converted using the Zymo EZ DNA methylation kit (Zymo Research, Irvine, CA) as specified by the manufacturer. Bisulfite-converted DNA quantity and completeness of bisulfite conversion were assessed for each sample using a panel of MethyLight-based real-time PCR quality control assays, as described previously [43]. Bisulfite-converted DNAs were then used as a substrate for the Illumina EPIC BeadArrays, using the method recommended by the manufacturer and first described in Moran, 2016 [44].

### DNA methylation analysis

All statistical analyses were carried out in R version 4.0.3. For all analyses, probes were removed in minfi [45] if: they had a detection *P*-value < 0.01; were cross-reactive; contained a SNP at the single base extension or CpG interrogation site at any MAF; were not present in a CpG context; were present on either sex chromosome. Given the cancer-related nature of some of the datasets analyzed, we also assessed probes in SeSAMe [41]. Probes that failed under default parameters were also removed prior to analysis. For all analyses, DMRs were generated using DMRcate [28] on beta values. A comparison of DMRs across conditions was then carried out by setting lambda = 1000 and minimum CpGs = 7. For Illumina HM450 data, C was set to 2; this value was doubled for EPIC array data to account for the increase in probe density on the EPIC array. Significant individual CpG sites were identified if they remained significant following Benjamini and Hochberg correction [46]. These sites were then agglomerated using the parameters established above, and a resulting Stouffer’s P value was determined. Following this, we performed a secondary false discovery rate (FDR) correction on Stouffer’s *P*-values generated from each DMR. Only DMRs with FDR < 0.05 and a mean absolute beta difference greater than 5% were deemed statistically significant. Overlapping DMRs were identified using bedR [47]. Pathway analysis was carried out on significant DMRs to search for enrichments in gene ontology (GO) terms [48] using the goregion () function of MissMethyl [49] with prior probability and fractional counts set to true.

For FAP and healthy control colon organoids, sex-stratified quantile normalization was preferred and performed under default settings [50]. A total of 744,851 probes were considered. To adjust for technical variation on the array, COMBAT was preferred, which was set to adjust for chip while retaining variation attributed to disease status in ChAMP [51, 52]. For DMR analysis across the 23 samples, gender and age were considered as additional covariates. For DNA methylation analysis of The Cancer Genome Atlas Colon Adenocarcinoma (TCGA-COAD) dataset [23], raw IDAT files were downloaded using TCGAbiolinks [53]. Functional normalization was used for array processing [54]. A total of 384,949 probes were used for downstream analysis. Given the imbalance between chip and sample status, we used ENmix [55] to estimate the number of principal components (PCs) needed to account for technical variation about the array. Five PCs accounted for 87.02% of variation about the array. These PCs and a dummy variable for sample pairing were used as adjustment covariates for DMR analysis. For ColoCare [24] analysis, data were downloaded from GEO [56], accession: GSE101764. Samples were excluded if they did not contain a matching pair, or if their matching pair was not present on the same chip. A total of 78 pairs were considered for analysis, which was carried out in the same manner as TCGA-COAD, with the exception that, instead of using ENmix, COMBAT [51, 52] corrections were performed on chip and sample location on the array prior to DMR analysis. Similarly, for GSE193535 [18], seven sample pairs were removed to balance demographic variable placement on the array. Downstream analysis was carried out in a manner similar to ColoCare. To infer the relative significance of the overlaps identified across studies and technologies, we performed an independent analysis of single CpG sites for each dataset. Beta values were processed using the same approach as for DMRs. For the identification of significant CpGs, the treat () function in limma was used [57], as previously [58] while specifying a 5% absolute log fold change of 5% in average betas between phenotypes. When comparing data across arrays, the number of FDR significant CpGs considered was restricted to sites that were only covered on both arrays following filtering in each dataset. All data have been made available to Gene Expression Omnibus and can be accessed through accession number: GSE19646.

### Mapping genes to CRC GWAS loci

We downloaded CRC GWAS index SNPs from the GWAS catalog and from Huyghe et al. [30, 59]. DMRs within CRC GWAS loci were defined as those with at least a single CpG overlapping a 1 MB interval centered on the index SNP. BedR was used to determine this overlap [47].

### Quantitative PCR (qPCR)

Genes were chosen for validation by qPCR if a corresponding DMR was identified in our initial FAP versus healthy colon organoid analysis and if the gene of interest replicated in both CRC DNA methylation datasets. To infer the likelihood that DMRs of varying significance levels would drive gene expression, genes were also not limited only to the most significant findings. In cases where a single DMR overlapped multiple genes, only the gene corresponded to the most significant individual CpG was considered (data not shown). A subset of five FAP and five healthy colon organoids were chosen for gene expression analysis. cDNA was synthesized from 2 μg of total RNA using the High-Capacity Reverse Transcriptase cDNA kit (Thermo Fisher). Quantitative real-time polymerase chain reaction was performed using the TaqMan Gene Expression Master Mix (Thermo Fisher) with TaqMan assays. *Glucuronidase Beta* (*GUSB*; Hs00939627_m1). The PCRs were performed in five FAP and five CTL lines, ran in duplicate and were analyzed using QuantStudio 5 (Thermo Fisher). Reactions were normalized using the control gene *GUSB,* and calculations were performed according to the 2^−ddCT^ method. Data were analyzed for statistical differences using a linear regression model on log-normalized values while adjusting for age and gender.

### Gene expression analysis of TCGA-COAD

Raw HT-Seq counts were downloaded from the R package TCGAbiolinks [53]. A paired regression analysis was performed in DESeq2 [60]. For significance testing, we employed a log_2_fold threshold of |0.25|. Given the specific nature of our analysis (12 genes were selected based on apriori data), multiple testing corrections were not considered.

## Supplementary Information

**Additional file 1: Figure S1**. Representative image of colon organoid lines with scale. For each distinct phenotype (represented by columns), images of three individual lines were captured (represented by rows).

**Additional file 2: Table S1**. Phenotype information for cohort.

**Additional file 3: Figure S2**. Representative images of three independent FAP (left) and healthy colon organoids taken at 100x magnification.

**Additional file 4: Table S2**. Summary of DMR analysis across all studies used in study that overlapped DMRs identified in FAP study. Positive "meandiff" corresponds to increased beta values (DNA methylation) in FAP (or tumor samples).

**Additional file 5: Table S3**. Summary of gene ontology analysis for FAP versus healthy colon organoids.

**Additional file 6: Table S4**. Summary of qPCR and RNA-seq analysis performed on selected gene targets. Positive fold changes and t values indicate increased expression in TCGA-COAD tumor and FAP colon organoids, respectively.

## Data Availability

Any novel, raw and pre-processed data used in this study have been uploaded to GEO [61], accession number: GSE19646.

## Abbreviations

CRC: Colorectal cancer
DMR: Differentially methylated region
FAP: Familial adenomatous polyposis
GEO: Gene expression omnibus
GO: Gene ontology
GWAS: Genome-wide association study
qPCR: Quantitative PCR
TCGA-COAD: The Cancer Genome Atlas Colon Adenocarcinoma
UVA: University of Virginia
