# Supplementary figures and images for: DNA methylation analysis of normal colon organoids from familial adenomatous polyposis patients reveals novel insight into colon cancer development
Source: Clin Epigenetics. 2022 Aug 23;14:104. doi: 10.1186/s13148-022-01324-5 (PMC9396789; doi:10.1186/s13148-022-01324-5)

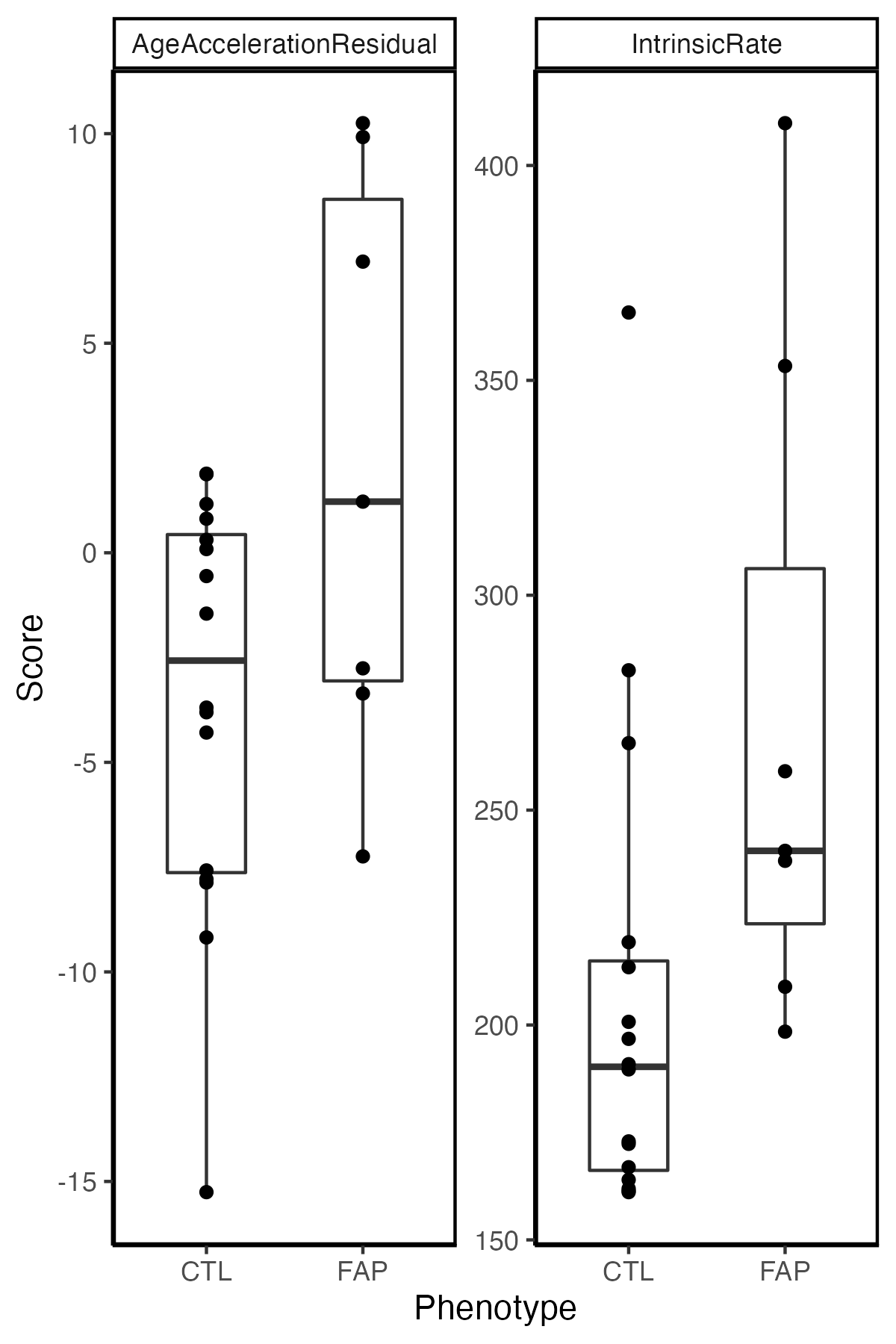

Supplement: Supplementary file 1 — Additional file 1: Figure S1. Representative image of colon organoid lines with scale. For each distinct phenotype (represented by columns), images of three individual lines were captured (represented by rows). [file 13148_2022_1324_MOESM1_ESM.tiff]

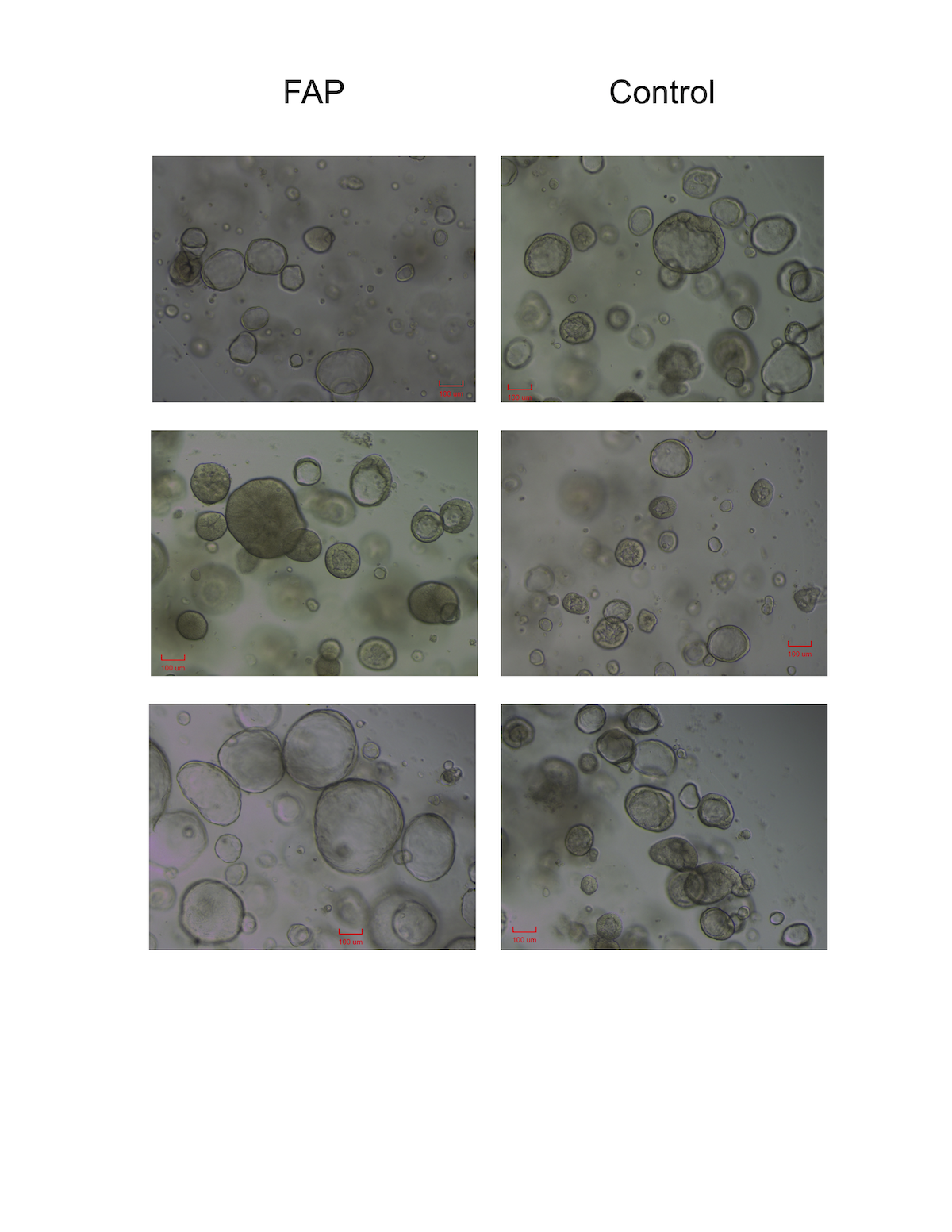

Supplement: Supplementary file 3 — Additional file 3: Figure S2. Representative images of three independent FAP (left) and healthy colon organoids taken at 100x magnification. [file 13148_2022_1324_MOESM3_ESM.tiff]
